# Activation and Repassivation of Stainless Steels in Artificial Brines as a Function of pH

**DOI:** 10.3390/ma12233811

**Published:** 2019-11-20

**Authors:** Emir Mujanović, Bojan Zajec, Tadeja Kosec, Andraž Legat, Stefan Hönig, Gerald Zehethofer, Gregor Mori

**Affiliations:** 1 Slovenian National Building and Civil Engineering Institute, Dimičeva ulica 12, 1000 Ljubljana, Slovenia; emir.mujanovic@zag.si (E.M.); bojan.zajec@zag.si (B.Z.); tadeja.kosec@zag.si (T.K.); Andraz.legat@zag.si (A.L.); 2 OMV Austria Exploration & Production GmbH, Protteser Strasse 40, 2230 Gaenserndorf, Austria; stefan.hoenig@omv.com (S.H.); gerald.zehethofer@omv.com (G.Z.); 3 Department of General, Analytical and Physical Chemistry, Montanuniversitaet Leoben, Franz Josef-Strasse 18, 8700 Leoben, Austria

**Keywords:** stainless steels, activation, repassivation

## Abstract

When planning oil wells with stainless steel components, two possible reasons for depassivation have to be considered—chemical depassivation caused by acidizing jobs and mechanical depassivation caused by various tools and hard particles. The study explores conditions causing chemical activation of investigated steels and circumstances under which repassivation occurs after activation. The main focus of the study is to determine, how quickly various steels can repassivate under different conditions and to find pH values where repassivation will occur after depassivation. The investigated steels were ferritic (martensitic or bainitic) in the cases of 13Cr, 13Cr6Ni2Mo, and 17Cr4Ni2Mo, austenitic in the case of 17Cr12Ni2Mo, and duplex (austenitic and ferritic) in the case of 22Cr5Ni3Mo. Potentiodynamic experiments were employed to obtain electrochemical properties of investigated steels, followed by immersion tests to find ultimate conditions, where the steels still retain their passivity. After obtaining this information, scratch tests were performed to study the repassivation kinetics. It was found that repassivation times are similar for nearly all investigated steels independent of their chemical composition and microstructure.

## 1. Introduction

In oil and gas wells the production of hydrocarbons is frequently stopped by plugging of the reservoir (limestone precipitation). Re-establishment of production is mainly done by acidizing jobs, where concentrated hydrochloric acid is fed into the borehole to dissolve plugs in the reservoir. When the flow of product is enabled again, a very low pH solution is produced until the pH rises back to the original pH in the well. As long as the pH of the fluids during and after acidizing jobs is below a certain threshold value, well components made of stainless steels (e.g., nozzles, gravel packs, in some cases also the tubing) are in the active state without passive layer. Additionally, in mature fields with decreasing well pressure, the production rates are maintained by increasing flow velocity in the tubing. This results in an increase of sand production and consequently in repeated local mechanical depassivation of stainless steel components by sand [1].

The protective properties of passive layers on stainless steel depend greatly on the conditions in which the steel is used and on alloying elements in the steel. Iron oxides found in the air formed passive layers, but are dissolved in acidic pH solutions, leaving a further enrichment of chromium oxides/hydroxides on the surface in the passive layer. Other alloying elements such as nickel, molybdenum, and nitrogen have both individual and synergistic effects on the protective properties of passive layers [2,3,4,5].

In a study on the role of corrosion resistant alloying elements in passivity [6], Hashimoto et al. reported on a decrease in passive current density of a 30Cr-2Mo steel compared to a 30Cr steel, due to MoO_2_ in the inner layer of the passive film acting as a diffusion barrier. The steel with molybdenum thus shows a passive current density two orders of magnitude lower than the steel without molybdenum in 1 M HCl [6].

The vast majority of repassivation studies was performed under potentiostatic control, using different depassivation techniques, ranging from micro-indentation [7] to scratching [5], guillotine tests [8] and others. When performing such experiments, the applied potential affects current measurement [9,10].

Of particular interest is a study discussing depassivation and repassivation behavior of stainless steels in NaCl solution investigated by micro-indentation, where Yamamoto et al. [11] reported that type 304, 312L, and 316L stainless steels all show similar repassivation kinetics despite having different chemical compositions. Partially in contrast to this, the beneficial effects of nitrogen on the repassivation behavior of type-304L stainless steel in chloride solution has been reported [12]. However, this beneficial effect was observed only at potentials more noble than −150 mV_SCE_.

Marshall and Burstein have discussed the influence of pH on repassivation of 304L stainless steel [13] in detail. They have found a potential, e.g., which represents the minimum potential at which a passive film can grow. While this potential is dependent on pH value, they claim that the pH of the electrolyte does not affect the kinetics of thickness growth of passive layers on the stainless steel, with the exception for very acidic electrolyte (pH 0).

When discussing passivity and steady state conditions of passivity, it is important to note that it is still unsure, what a true steady state in passivity in fact is. In their publication [14], Burstein and Daymond have shown that it is possible to achieve extremely low passive current densities by polarizing a sample of 316L stainless steel, while applying temperature sweeps to the electrolyte (in their case nine cycles between 30 and 85 °C). Even then the authors were unsure, whether an actual steady state (final state) of passivity has been reached, despite a current density as low as 0.04 nA/cm^2^ has been achieved.

The present study is not focused on reaching a true (final) steady state of passivity. Experiments were designed to identify a reasonable time scale for performing the tests and to define a current density value, where a certain passivity is reached.

The goal of this paper is to find criteria, when a stainless steel can be claimed to be in the passive state and under which conditions this passive behavior is reached. Three types of experiments have been done, namely cyclic polarization measurements to get a first electrochemical characterization of the materials, immersion tests at different pH values to identify type of attack for different materials and following scratch tests to study repassivation kinetics. Experiments in the present study were performed with five different stainless steels in 30,000 mg/L Cl^−^ containing brine, deaerated at 30 °C.

The results shall improve the understanding of the effect of chemical and mechanical depassivation on the lifetime of oilfield components made of stainless steels.

## 2. Materials and Methods

Chemical composition of materials investigated is given in Table 1. A wide range of alloying elements has been chosen to get a deeper insight into activation and repassivation behavior of stainless steels. Beside conventional alloys such as a 13% chromium martensitic stainless steel (labelled as 13Cr in this work) the austenitic stainless steel 17Cr12Ni2Mo (316L) and the duplex stainless steel 22Cr5Ni3Mo (2205), also modified alloys with 13 and 17% chromium with additional amounts of nickel and molybdenum (labelled 13Cr6Ni2Mo and 17Cr4Ni2Mo) have been chosen to be tested. The lowest alloyed material 13Cr is at the very low edge of the composition range of passive steels, while duplex steel 22Cr5Ni3Mo has a PREN (see Equation (1) [15,16]) of 34.6 as the highest alloyed investigated material.

PREN = %Cr + 3.3%Mo + 16%N.(1)

Namely, pitting resistance equivalent number (PREN) is a predictive measurement of a stainless steel’s resistance to pitting corrosion based on its chemical composition. In general a higher PREN value yields a more resistant stainless steel to pitting corrosion initiated by halide ions (in most cases chloride ions).

Stainless steel samples were prepared by wet grinding with #600 abrasive SiC paper and subsequent storage for at least 24 h in a desiccator to allow repassivation.

Cyclic polarization measurements have been performed in a solution with 30,000 mg/L Cl^−^ added as NaCl. The solution was purged with 1 bar of CO_2_ for 1 h prior to and during testing to adjust a pH of ~4.3, which is close to theoretical depassivation pH according to the Pourbaix diagram of chromium in water [17]. Additionally the amount of dissolved oxygen was measured in several tests, which was shown to be less than 100 ppb. For the reference electrode a saturated calomel electrode (SCE) was used, the counter electrode was made of an annealed platinum sheet. Before scanning at a scan rate of 200 mV/h the open circuit potential (OCP) has been measured until it was stable and at least for 1 h. Potential scan started at 100 mV below OCP. Scan rate was reversed when reaching an anodic current density of 1 mA/cm^2^. Polarization scans were done only once per each steel type since breakdown potentials show a plausible increase with an increase of PREN, and width of passive ranges show also a steady increase with an increase of PREN.

Immersion tests were done in beakers for 24 h in 30,000 mg/L Cl^−^ added as NaCl at 30 °C and at different pH values, namely at pH 5, pH 4, pH 3, pH 2, pH 1, and pH 0. pH was adjusted with addition of HCl. All electrolytes were purged with 1 bar argon for 1 h prior to and during exposure, ensuring a dissolved oxygen level of less than 100 ppb. The testing time was 24 h. Immersion tests were done once per each steel type with a single specimen in each solution. In case of implausible results a second test was performed and if the test result of the second test differed from the first, a third test was done. The two identical test results are reported in this study.

Scratch tests were done by scratching the sample with a diamond tip of a Vickers hardness indenter. The scratch test setup is spring loaded, causing a constant force on the diamond tip once the latch is released. In cases where the scratch was not uniform in depth and the diamond tip “jumped” across the surface the result was not taken into consideration and the measurement was repeated, leading to similar depth and size of scratches (scratched surface was between 0.65 and 0.8 mm^2^ in all cases). The measured current was then divided with the measured and calculated scratched area yielding the current density for each individual experiment. To avoid potentiostatic control during scratch tests and to attempt to apply field conditions, it was decided to perform scratch experiments by using two identical stainless steel electrodes at open circuit potential, measuring the current flow between them during scratching and repassivation. The two identical electrodes of the investigated material have been immersed in the solution for 20 h, to allow a steady state to be reached (area of each electrode was 19.6 mm^2^). The scratched electrode in such a setup is usually called working electrode 1 (WE 1), while the unscratched electrode is called working electrode 2 (WE 2). The two electrodes were in contact via a PTFE insulated nickel wire during the immersion time prior to performing the scratch. A zero resistance amperemeter was used without applying a potential—both samples were at open circuit potential (OCP) in the solution. For the reference electrode again a SCE with a potential of 241 mV_SHE_ was used.

**Figure 1:**
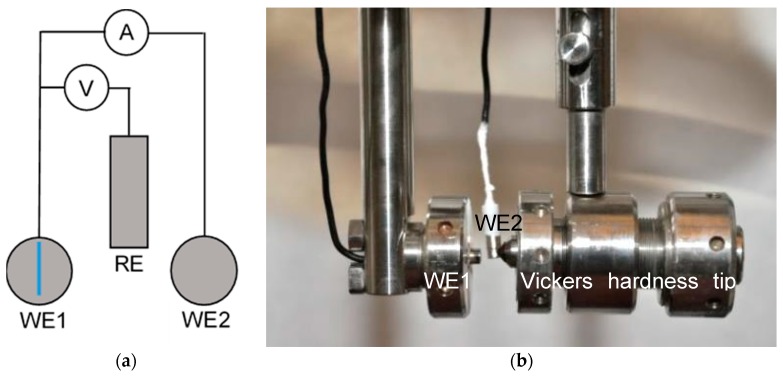
Schematic (**a**) and experimental setup (**b**) of scratch test; WE1—working electrode 1 (scratch is blue vertical line in (**a**)); WE2—working electrode 2 (not scratched); RE—reference electrode.

The procedure is described elsewhere [18], with the addition of 1 bar argon purging 1 h prior to and during experiment, ensuring a dissolved oxygen content below 100 ppb. The experimental setup of the scratch test is shown schematically in Figure 1 along with a photograph of the two identical samples with the scratching diamond tip. In contradiction to earlier works [18] data were logged at a higher frequency of 500 Hz with an HRU/ZRA system made by IPS, Germany. Current between the two identical electrodes was divided by the scratched area (between 0.65 and 0.80 mm^2^), which has been determined by optical microscopy under consideration of a diamond tip angle of 120°. Results of scratch tests were drawn as double logarithmic diagrams log(i) vs. log(t), where i is the current density and t is the time after the scratch. The scratches as an average were produced within 26 ms with a scatter of ±0.006 s. Some scratch events were recorded with a high speed camera and the scratch time observed was compared to the current transient before the current decay, which was shown to be the same time (±0.001 s). Scratch tests of several steels have been repeated up to three times and representative repassivation curves are shown in this work.

## 3. Results and Discussion

**Figure 2:**
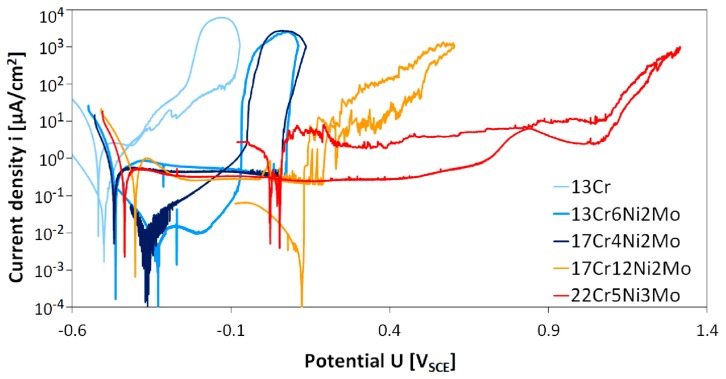
Potentiodynamic measurements of investigated stainless steels in 30,000 ppm Cl^–^ (as NaCl), 30 °C, 1 bar CO_2_, 200 mV/h.

Results of cyclic polarization measurements of five different stainless steel grades are given in Figure 2. Electrochemical data derived from the cyclic polarization tests are given in Table 2.

It can be observed from cyclic polarization (CP) curves that passive regions widths differ in relation to different stainless steel grades. Material 13Cr shows a very narrow passive range with a width of slightly more than 100 mV at a high passive current density of 2.2 to 2.5 µA/cm^2^. Breakdown potential (*E*_b_) is lower compared to all other steels at –400 mV_SCE_. The next two modified chromium steels 13Cr6Ni2Mo and 17Cr4Ni2Mo show similar behavior. Compared to lower alloyed 13Cr, these two materials exhibit a slightly higher corrosion potential, a wider passive range of more than 500 mV, and a lower passive current density (80% lower than 13Cr). Austenitic stainless steel 17Cr12Ni2Mo shows a similar passive current density and corrosion potential like the two Ni and Mo containing ferritic chromium steels 13Cr6Ni2Mo and 17Cr4Ni2Mo. The breakdown potential E_b_ is higher at 189 mV_SCE_ than for the two modified chromium steels 13Cr6Ni2Mo and 17Cr4Ni2Mo (115 to 130 mV higher).

In contradiction to all other steels, duplex steel 22Cr5Mo3Ni showed tiny pits after the experiment. This steel showed the highest breakdown potential *E*_b_ = 1063 mV_SCE_ of all investigated steels. Corrosion potential and passive current density were very similar to modified chromium steels and the austenitic steel. The passive range was much wider with a total width of 1500 mV.

Results of immersion tests are presented in Table 3.

Immersion tests consisted of 24 h of exposure of five different steel grades in different pH solutions. Material 13Cr was the only one to show pitting at pH 4, while all other materials remained not corroded. At pH 3 13Cr showed uniform corrosion, while all others started to pit. At pH 2 the modified steel 13Cr6Ni2Mo also showed uniform corrosion, while the higher alloyed materials still showed pits in a passive state. At pH 1 the 17Cr4Ni2Mo steel was uniformly corroded as well. At pH 0 all materials were completely depassivated after 24 h of exposure to the corrosive solution and showed uniform corrosion. For all materials a decrease of pH of the test solution starting from the “no corrosion” range, resulted in a localized attack, namely “pitting corrosion”. Under pitting conditions a localized breakdown of the passive layer occurs, resulting in holes in the passive layer and further yielding to localized and statistically distributed pit formation. Anodic pits are always surrounded by cathodic zones that can still maintain passivity, while inside the pits pH is further decreased and chloride concentration increases. When further decreasing the pH of the test solution until the passive layer is completely dissolved, “uniform corrosion” occurs. Under these conditions three valent chromium in the passive layer is oxidized to six valent chromate ions and the main constituent of the passive layer (chromium oxide/hydroxide) dissolves. Below these pH values the passive layer is no longer stable and the stainless steel dissolves in the same way as an active (non-passivating) metal.

**Figure 3:**
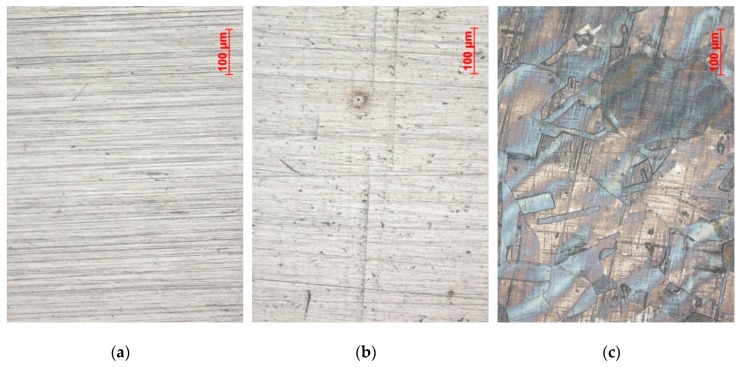
Optical image of corrosive attack of austenitic stainless steel 17Cr12Ni2Mo after exposure to different pH in 30,000 ppm Cl^−^ at 30 °C for 24 h, pH adjustment with HCl, (**a**) pH 4: no corrosion, (**b**) pH 3: pitting corrosion, (**c**) pH 0: uniform corrosion.

Figure 3 shows the appearance of austenitic material 17Cr12Ni2Mo after tests at different pH values. No sign of corrosion was observed in pH 4 (Figure 3a). In pH 3, pits started to form during the 24-h exposure (Figure 3b). They were of 10 ± 4 µm diameter in average. Uniform corrosion resulted in pronounced grain etching after the experiment on austenitic steel, as shown in Figure 3c. Ferritic steels were covered in dark corrosion product residue. Duplex stainless steel had one phase preferentially corroded when exposed to pH 0 and uniform corrosion was detected.

**Figure 4:**
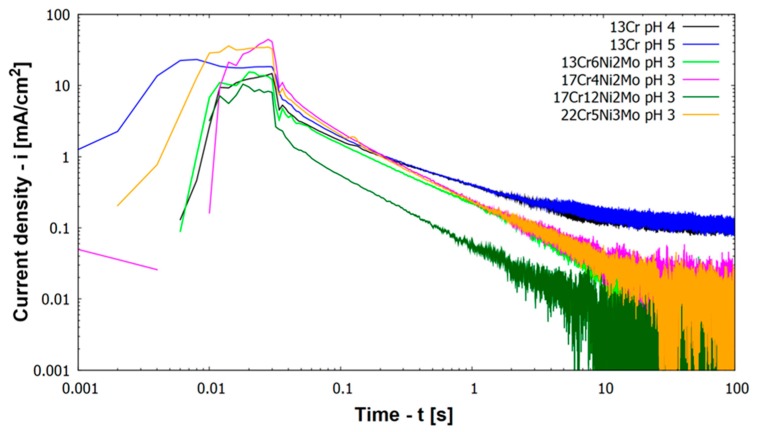
Current density vs. time during scratching and repassivation of investigated materials in 30,000 ppm Cl^–^ at 30 °C (scratch ended after 0.03 s).

Figure 4 depicts results of repassivation curves for higher alloyed materials (including all materials except 13Cr) at pH 3. Figure 4 includes also results for 13Cr at other pH values, namely pH 4 and pH 5.

All higher alloyed materials with Mo show repassivation by reaching a current density below 0.01 mA/cm^2^ within less than 100 s at pH 3 (Figure 4). Kinetics of repassivation were the same for all these higher alloyed CrNiMo stainless steels, which can be seen by the parallel log(i) vs. log(t) plots in Figure 4. Only austenitic material 17Cr12Ni2Mo shows a lower log(i) vs. log(t) slope/curve compared to other materials. Regarding the height of the activation peak this was smallest for austenitic material 17Cr12Ni2Mo.

**Figure 5:**
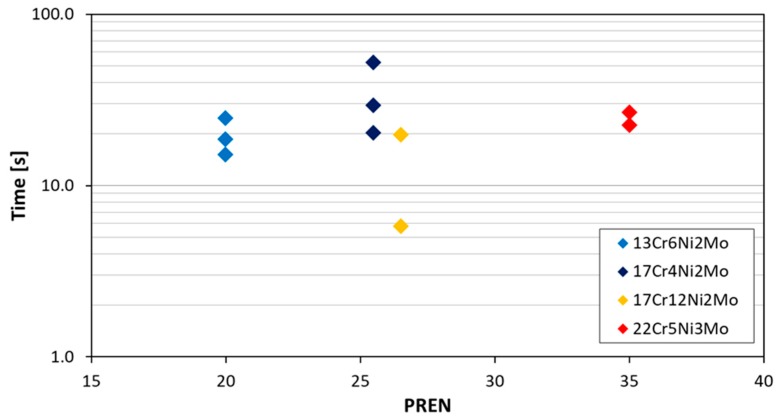
Time from scratching until reaching a current density of 0.01 mA/cm^2^ (roughly equal to a corrosion rate of 0.1 mm/year) for investigated alloys in 30,000 ppm Cl^–^ at 30 °C at pH 3 as function of alloying content.

Figure 5 shows groups of measurements for four different steels, where the time to repassivate is below a corrosion current density of 0.01 mA/cm^2^ (corresponding to roughly 0.1 mm/y corrosion rate). As it can be observed from the results, the repassivation time is independent of alloying content (PREN) for the higher alloyed materials. The shown repassivation time is between 6 and 50 s at a scatter of half of an order of magnitude. The value of 0.01 mA/cm^2^ has been chosen since it is below the active corrosion or pitting current density but still above steady state passive corrosion current densities, which are around 0.001 mA/cm^2^. So it can be assumed that the material has made a large step toward this steady passive state (represented by a corrosion current density of 0.001 mA/cm^2^). The steady passive state is considered as where the metal reaches in the passive range a constant corrosion current density. This state still includes some oxidation and reduction on the surface of the material. Reaching a passive state without any identifiable activity can last much longer as shown by Göllner et al. [19]. They found by the use of electrochemical noise an increased activity of an activated specimen even after 100 h in humid air with 95% relative humidity and also after 1000 h in laboratory air at 45% relative humidity.

In contradiction to the higher alloyed steels at pH 3, the behavior of 13Cr at pH values 4 and 5 is different (Figure 4). Repassivation characteristics shifted from a mainly linear behavior toward a degressive behavior. 100 s after scratching the specimen 13Cr stainless steel remains at an anodic current density of 0.1 mA/cm^2^ (100 µA/cm^2^), which is one order of magnitude higher than for the other materials and 2 orders of magnitude above passive corrosion current density (compare with Figure 2). The lack of repassivation below a corrosion current density of 0.01 mA/cm^2^ is the reason, why material 13Cr cannot be drawn in Figure 5.

**Figure 6:**
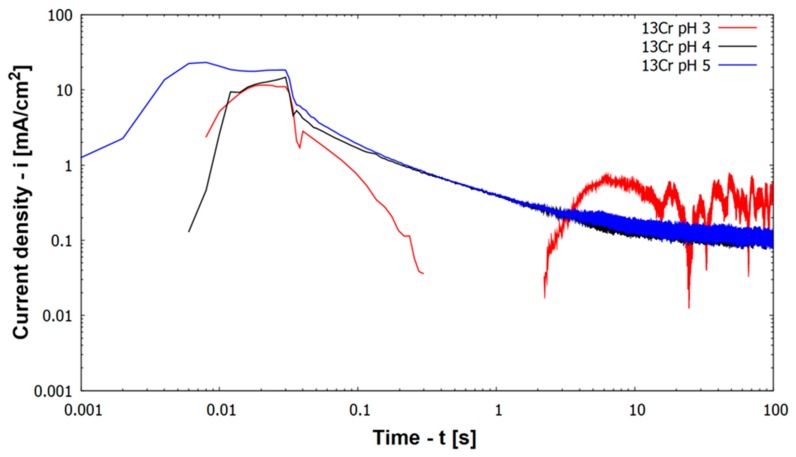
Current density vs. time during scratching and repassivation of 13Cr stainless steel in 30,000 ppm Cl^–^ at 30 °C at different pH.

Figure 6 shows log(i) vs. log(t) plots for 13Cr at pH 3, 4, and 5. Results for pH 4 and 5 have already been described, when a fully passive state has not been reached. At pH 3 material 13Cr activates already prior to scratching as shown in immersion tests. Immediately after scratching the material starts to repassivate and there is a sharp decrease in anodic current density. After 0.3 s, the scratched sample even becomes cathodic in comparison to the unscratched sample until after 2 s it turns anodic again. This anodic corrosion current density increases and remains at a value between 0.1 and 0.8 mA/cm^2^. There are strong fluctuations of corrosion current density, which stays for the active sites on both samples in the electrolyte, the scratched and the unscratched one. During the next 100 s the scratched specimen remains on the more active one.

**Figure 7:**
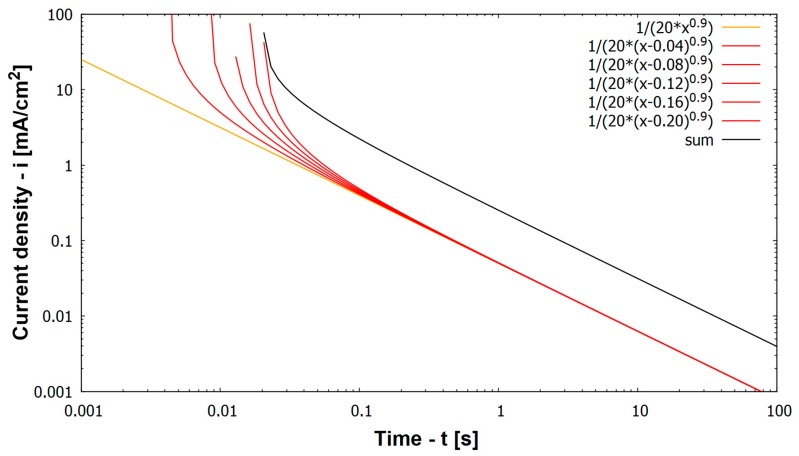
Simulation of repassivation behavior for a full scratch (black line) and for discrete and time shifted parts of the scratch by using Equation (2) (sum of red lines is black line), orange line indicates instantaneous start of repassivation.

Mathematical description of oxide layer formation on a metal has first already been described by Stern [20]. He found a linear log(i) vs. log(t) behavior. Other authors also have found this relation (Equation (2) (orange line in Figure 7) [9,21,22]).

i(t) = a·t^−α^ or log i(t) = −α·log t + k, (2)

i(t) is the corrosion current density as function of time after scratching, a is constant (and k = log a is as well constant), t is the time after the scratch and α is the time constant of repassivation. Based on Equation (2) a model of repassivation current density of a whole scratch has been generated. Since scratching needs approximately 26 ms of time and the bare scratched metal immediately starts to repassivate at a high rate (see steep negative slope of current densities immediately after scratching in Figure 4), it is wrong to assume the scratch as one single activation process over its whole length followed by repassivation throughout its length. Instead, repassivation at the initiation point of the scratch already propagates substantially, while the scratch is still introduced at a later stage into the specimen. Consequently the scratch has been divided into five sections (with a time shift t_0_, or in this case 4 ms between each). The five sections of the scratch represent different stages of repassivation, (Equation (3) red lines in Figure 7) represented by the red lines in Figure 7, which are shifted by 4 ms from the preceding one.

i(t) = a·(t − t_0_)^−α^ (3)

The five sections of the scratch are shown by red lines in Figure 7. Note that scratching takes only 26 ms while repassivation takes a time of several seconds. Therefore the five different sections of the scratch just represent different stages of repassivation during the very first 100 ms (0.1 s) after starting the scratch. After more than 100 ms all sections show the same current densities representing the same stadium/propagation of repassivation. This is due to the fact that (1) in the model a time shift of 4 ms between the different sections of the scratch can no longer be shown in the logarithmic time axis and that (2) in the experiment a time shift of a few ms between two sections of the scratch is no longer relevant for the measured corrosion current density after some 100 ms and more. The sum of the five red lines is the black line that represents the current density of the whole scratch during a scratching event. The model describes the experimental findings for log i(t) vs. log t relation very well. The orange line in Figure 7 represents instantaneous start of repassivation. However, as the scratch event is in fact represented by several such lines at different times (Equation (3)), all of these discrete points have to be added to describe the current density response that is created by a scratch event (black line in Figure 7).

The most important outcome of the log (i) vs. log t plots is that all materials show the same or rather similar kinetics of repassivation. This may be additionally seen in Table 4, which details the α values obtained from each repassivation curve according to Equation (3) at times between 0.1 s < t < 1 s. If a current density of 5 mA/cm^2^ is assumed at time of scratch ending (t = 0.03 s), the time needed to reach 0.01 mA/cm^2^ current density is calculated to be 49 s under a α = 0.8 regime, while under a α = 1 regime the time to reach that current density is only 10 s. The negative slopes of all curves in Figure 4 and Figure 7 remain for this very similar rate of repassivation independent of alloying elements. Only 13% Cr steel in Figure 4 show at pH 4 and pH 5 a less negative slope, which corresponds to no sufficient repassivation for this steel in these environments. For practical applications of stainless steels in oil and gas production an acidizing job (chemical depassivation) will result in activation of well components made from stainless steel. Based on the results above, repassivation will take place within short times (within 100 s), when the pH of the produced brine rises above the threshold pH. In case of mechanical depassivation by sand particles the degree of attack is determined by the frequency of sand impacts. Once the passive layer is damaged it needs up to 100 s to build up again. In case within this time the next sand grain impacts the same surface area, the material will remain active.

It is still not clear, whether there are conditions in the investigated corrosion systems (steel—electrolyte conditions), where the electrolyte can maintain passivity as long as there was no mechanical activation and yet does not allow for repassivation of the material after it has been scratched. Such a state could result in substantial loss of lifetime of oilfield components and shall be avoided.

Future investigations will focus on more exact identification of activation and repassivation pH as a function of alloying content in steels. For this a flow through cell is currently established that can be loaded with up to eight materials simultaneously because of the use of a multichannel potentiostat and electrochemical measurement device (as described by Linhardt et al. [23]).

## 4. Conclusions

The repassivation properties of five different stainless steel grades were evaluated. Three types of experiments were used: cyclic polarization curves, immersion tests, and repassivation experiments in order to distinguish between stainless steel properties in high chloride low pH and low oxygen environment. The following conclusions can be drawn from the present work:

- Different steel grades with different alloying contents show activation and repassivation at different pH values.
- As long as repassivation under certain conditions is possible, it takes place within the same time range for different alloys and microstructures.
- In case a stainless steel has a too low content of alloying elements for the given electrolyte conditions, it cannot repassivate quickly and corrosion current density remains high.

## Figures and Tables

**Table 1:** Chemical composition and pitting resistance equivalent number (PREN) of investigated materials (wt.%).

Material	Material Designation	C	Si	Mn	P	S	Cu	Cr	Ni	Mo	N	PREN
13Cr	410	0.19	0.32	0.52	0.013	0.004	-	12.4	0.18	0.01	0.022	12.8
13Cr6Ni2Mo	-	0.01	0.16	0.36	0.016	0.002	0.05	12.6	5.9	2.2	0.007	20.0
17Cr4Ni2Mo	-	0.03	0.26	0.33	0.014	0.003	0.82	16.9	3.7	2.4	0.035	25.4
17Cr12Ni2Mo	316L	0.01	0.45	1.87	0.016	0.001	-	17.2	11.6	2.3	0.063	25.8
22Cr5Ni3Mo	2205	0.03	0.53	1.79	0.018	0.003	-	22.1	5.4	3.3	0.101	34.6

**Table 2:** Electrochemical data from potentiodynamic measurements of investigated materials in 30,000 ppm Cl^–^ at 30 °C, 1 bar CO_2_, 200 mV/h.

Material	*E*_corr_ (mV_SCE_)	*i*_corr_ (μA/cm^2^)	*E*_b_ (mV_SCE_)	*E*_b_ − *E*_corr_ (mV)	*E*_rep_ (mV_SCE_)	*E*_rep_ − *E*_corr_ (mV)	*i*_pass_ (μA/cm^2^)
13Cr	−518	1.45	−401	117	−422	96	2.20–2.46
13Cr6Ni2Mo	−464	0.28	74	538	−69	395	0.27–0.65
17Cr4Ni2Mo	−468	0.29	57	525	−128	340	0.31–0.44
17Cr12Ni2Mo	−402	0.35	189	591	130	532	0.20–0.28
22Cr5Ni3Mo	−435	0.30	1063	1498	59	494	0.24–0.34

*E*_corr_—open circuit potential; *i*_corr_—corrosion current density; *E*_b_—breakdown potential; *i*_pass_—passive current density; *E*_rep_—repassivation potential.

**Table 3:** Type of corrosive attack on investigated materials as function of pH in 30,000 ppm Cl^–^ at 30 °C for 24 h. Legend: u—uniform corrosion, p—pitting corrosion, n—no corrosion.

Material	pH 0	pH 1	pH 2	pH 3	pH 4	pH 5
13Cr		u	u	u	p	n
13Cr6Ni2Mo	u	u	u	p	n	
17Cr4Ni2Mo	u	u	p	p	n	
17Cr12Ni2Mo	u	p	p	p	n	
22Cr5Ni3Mo	u	p	p	p	n	

**Table 4:** α Values obtained from scratch tests in the linear regime found in log(i) vs. log(t) diagrams, between times t = 0.1 s and t = 1 s.

Material	Scratch 1 α	Scratch 2 α	Scratch 3 α	Mean α
13Cr6Ni2Mo	0.8	0.83	0.97	0.867
17Cr4Ni2Mo	0.78	0.86	0.96	0.867
17Cr12Ni2Mo	0.86	0.98	-	0.92
22Cr5Ni3Mo	0.78	0.93	-	0.855
